# Statistical Reports on the Health of the Navy, for the Years 1830-1-2-3-4-5-6. (South American, West Indian and North American, Mediterranean, and Peninsular Commands.)

**Published:** 1841-01

**Authors:** 


### Art. XI.

*Statistical Reports on the Health of the Navy, for the years* 1830-1-2-3-4-5-6. (*South American, West Indian and North American, Mediterranean, and Peninsular Commands.*)—*London,* 1840. pp. 323.

We have already* presented our readers with a detailed account of the valuable reports on the diseases of the army, prepared with so much care and accuracy under the superintendence of Major Tulloch. We now present them with an abstract of the first of a series of statistical reports on the health of the navy, which we owe, in the first instance, to the public spirit of Sir William Burnett, the distinguished Physician-general, to whom the medical department of the navy is under so many obligations, and secondly, to the zeal and industry of Dr. John Wilson, R.N.; and in doing so we cannot but congratulate our profession on the valuable addition thus made to the statistics of health and disease.

In a short prefatory note we are informed that the reports were commenced as early as the year 1836, but that, owing to the sudden death of the officer first appointed to the duty, and the defective arrangements first adopted, it became necessary to begin the whole work *de novo,* and to examine sources of information which had been previously neglected. The officer first employed in drawing up these reports had omitted to trace disease from the ships to and through the hospitals, and had employed an erroneous method of estimating the mean force of the several squadrons. Dr. Wilson carefully avoided these and similar sources of error, and with the able assistance of Mr. George Mackeson, whose services he gratefully acknowledges, has succeeded in giving a faithful report of the health of the navy.

The materials from which these reports have been compiled are furnished by the surgeons and assistant-surgeons in charge of the health of ships' companies. These officers are required to transmit to the physician-general of the navy the following documents, namely, a daily sick-book, monthly or three-monthly nosological returns, and a journal of medical and surgical practice. In the daily sick-book are inscribed in separate columns the patient's name, age, quality, nature of disease or injury, date of discharge from the list, and the issue of the complaint, whether in cure, removal to hospital, invaliding, or death. This journal is transmitted annually. The monthly or quarterly nosological returns are transmitted every month from ships on the home station, and every quarter from those on foreign stations, and still more frequently during the prevalence of unusually severe and fatal diseases. They consist of tabular abstracts of the contents of the daily sick-book; and the diseases are arranged according to Cullen's system of nosology. The journals of medical and surgical practice contain detailed accounts of the symptoms, treatment, and result of diseases, trace them to their source, account for them when practicable, and suggest appropriate remedies. "It is also directed that states of weather, degrees of temperature, the general interior economy of the ships, and whatever else may appear to conduce to health or to induce disease be specified." In addition to these re-

* Brit. and For. Med. Review, No. XV. p. 221; and No. XVII. p. 63.

turns from the medical officers in the charge of ships, there are also returns from hospitals, naval,* military, and colonial,† and from sick quarters.‡

We have been thus minute in describing the sources from which the materials for these statistical reports have been obtained, that our readers may be in possession of the means of judging of their probable completeness. It is the tracing of the several sick and injured seamen, entered in the numerous ship returns, to and from the hospitals or sick quarters to which they have been removed, which constitutes the principal labour of such statistical reports as those which we have now before us; and when we state that the materials have had to be collected from ninety thick folio volumes we shall give some idea of the labour which has been expended. With all the care which could be bestowed on the preparation of these documents, it was impossible to avoid one source of error, we mean that arising from the sickness or death of the medical officers in charge of ships. This of course led to the occasional omission of the several returns to which we have alluded, an omission which could only be imperfectly supplied by stating the average of the force, disease, and mortality of the single ship thus deprived of its medical officers, from the returns of the squadron to which she belonged. Dr. Wilson expresses himself confidently as to the accuracy, ("if not absolute to a unit, at least a very close approach to it,") of the reports in respect of the rate of mortality, but he does not feel the same confidence with regard to invaliding. It appears, indeed, to be absolutely impossible to avoid error in tracing a sick seaman from the ship in which he first fell ill to some foreign hospital, thence to the packet which is to convey him home, thence again to the receiving ship which is to send him to the home hospital or sick quarters, and lastly, from the hospital or sick quarters to his death or discharge, or back to active service, should he be so fortunate as to return to it.

In consequence of the difficulties which stood in the way of a complete statistical report of the health of the navy, it is necessary to employ some caution in estimating the value of the facts established by the report. The same caution is required when we attempt to estimate the influence of climate in the production and cure of disease. In doing so, many circumstances peculiar to the naval service must be borne in mind, of which the principal are the short term (rarely more than from three to four years), during which ships remain in commission, the brief period of sojourn in any particular spot, the great territorial extent of many of the commands, and the consequent frequent and extraordinary change of climate to which the crews are subject. Add to these circumstances the effect, not of climate merely in the ordinary sense of the term, but of the variations of soil and of the emanations arising from it, as well as of food and water supplied by the several stations, and we have a multiplicity of influences brought to bear on the bodies of our seamen in quick

* There are five navy hospitals, namely, at Portsmouth, Plymouth, Malta, Jamaica, and Bermuda, and two marine hospitals, those of Chatham and Woolwich.

† Sick seamen and marines are received into all military and colonial hospitals, in such places as do not contain naval hospitals.

‡ There are fifty sick quarters for the reception of sick and injured seamen and marines, all of which are in the United Kingdom.

succession, the particular value of each of which it is altogether impossible to define or fix. These observations apply more especially to chronic diseases, whether primary or the consequence of acute affections: acute diseases themselves create little embarrassment, and can be commonly traced with care and certainty to the localities which produce them. Thus the various forms of fever, elephantiasis, dysentery, or acute inflammation in general may be fairly ascribed to the influence of the spot in which they arise. On the other hand, "in consumption and other insidious diseases of the lungs, and in various chronic affections of the liver and bowels, it is often impossible to detect their origin, and consequently to ascertain the portion of the command to which they should be ascribed; it is often difficult to determine whether, and to what extent, change of place, within the limits of the command acts beneficially or detrimentally in such cases." Of the great extent of some of the commands, we may take the West Indian and North American as an example. "It extends from the equator to the 60th degree of north latitude, and embraces every British possession, continental and insular, from Guiana to Cape Charles on the coast of Labrador." A command so extensive, embracing as it does so great a variety of climate, occasions much uncertainty in all our attempts to trace diseases to their exact cause; but there can be no doubt of the beneficial influence of the frequent changes from high to low latitudes, and from unhealthy to healthy climates on the health and efficiency of the ships' crews. Thus we learn from the report that "after a certain period of service in the West Indies, there is often so much diminution of organic force as to render men of little original power or advanced years incapable of laborious duty, and to make them more susceptible of disease, both acute and chronic. In these cases, and after attacks of severe disease, when convalescence is slow and uncertain, a run to Halifax or the Gulf of St. Lawrence acts like a charm. Health and strength, and with them spirits, are acquired at a rate scarcely to be believed by those who have not witnessed their progress, and many who but for the change must have sunk, or been sent to England, have been restored in a short time to full efficiency within the limits of the command, whether they remained in the northern or returned to the southern division of it."

It is necessary to observe that, in the returns of the sick and hurt, the patients are not classed, but all, from the admiral in command to the youngest boy in the ship, are comprehended in one table, in which the diseases and injuries affecting them are arranged, but in which neither the rank nor line of duty of the individuals composing the ship's company is specified. Great difficulties are obviously placed in the way of any classification which should at the same time describe the various influences to which the several classes are subject, and without such description no information of any value could be obtained. Aware of these difficulties, Dr. Wilson has very judiciously abstained from those minute classifications which only lead to imperfect and erroneous conclusions, and throw discredit on a mode of investigation to which we must owe almost all the valuable information yet to be gleaned from observation. For similar reasons, all mention of age and its influence on disease has been omitted from the report, as the age of seamen is not readily obtained where so many motives to false statements exist.

The introduction to the report, from which the foregoing abstract has been taken, enters into minute detail as to some other points necessary to be known and borne in mind in order to form a correct estimate of the report itself. Amongst these, the important article of *diet* holds a prominent place, and a minute and detailed account is given of the different kinds of provisions with which the ships are supplied. It is sufficient for our purpose to observe that the naval rations are abundant in quantity and excellent in quality; the several articles of food being of the very best kind which can be procured, and care being taken to vary the diet as much as possible. After fourteen days' use of salt food, lemon-juice with an additional quantity of sugar is used as an antiscorbutic. The introduction of tea, coffee, and cocoa, as substitutes for a moiety of the spirits formerly allowed, is not the least among the many important improvements which have been gradually adopted in the dietary of our ships of war. The diet of the sick has kept pace in improvement with that of the healthy portion of the service, and a glance at the list of articles allowed to patients in the royal naval hospitals and marine infirmaries, and as far as practicable on board ships, will show that nothing is omitted which can possibly promote the recovery of the sick.

In another article of not less importance than food, an improvement fully as great has of late years taken place; we mean the supply of *water*. The substitution of iron tanks for wooden casks, partially introduced into the navy before the year 1815, but since that period universally employed, has furnished an abundant supply of pure water, and has thus added materially to the comfort and salubrity of our ships of war.

A few words on the modes of employment and berthing of the men, and on the cleaning and ventilating of the ships, will conclude what we have to say by way of introduction, to the subject-matter of the reports.

Seamen and embarked marines, with the exception of those employed as sentries, are divided into two watches, which work the ship by turns. Each party has four hours' duty and four hours' rest; in other words, is four hours on deck and four hours below, excepting the four hours between four and eight o'clock at night, which are divided into two half, or, as sailors call them, dog-watches. The effect of this arrangement is to alter constantly the periods of labour and rest to each party, so that the men who have the first watch one night have the second watch the next night, and so on. "In this way, though there is a sufficient time for rest, it is never long continued, and sleep is broken into short periods. Whatever effect such a division of labour and rest may have on constitutional vigour, though it is perhaps the best that can be devised, it probably contributes much to the frequency of some affections, such as catarrh and rheumatism, to which seamen are so subject. At midnight, or four o'clock in the morning, the men relieving the deck rush from their beds into the open air, often very inadequately covered, perhaps perspiring profusely, and pass in an instant from a highly-heated and debilitating to, it may be, a really cold, always to a comparatively cold, atmosphere. In such circumstances it is to be expected that catarrhal, rheumatic, and other affections, traced to sudden reductive changes of temperature, should be numerous. These remarks apply chiefly to ships at sea. In port, the number of men kept on deck lessens with the increasing safety of the anchorage, till, in ships moored in secure harbours, all hands, excepting the officer, petty officers, and sentinels in charge, may pass the whole night in bed."

Seamen and marines mess and sleep on the middle and lower decks in first-rates, and on the lower deck in all vessels under first-rates. Ships of the line, moreover, have a deck (the orlop deck) between the lower gun-deck and the holds, whilst the lower deck in frigates and all smaller vessels is immediately above the holds. The means of ventilation in ships of the line are consequently far superior to those enjoyed by smaller vessels; for in the former there are ports which can be kept open in moderate weather, whilst in the latter there are only small apertures (scuttles) close to the water, which cannot be kept open except in an absolute calm at sea and very fine weather even in harbour. In all ships, whatever their size, in consequence of the great number of men on board, there must be great crowding between decks, and this crowding is greater as the vessel is smaller; in this respect then, as well as in point of ventilation, at least by means of the ports, ships of the line have the advantage over smaller vessels, but it may be doubted whether the interior ventilation through the hatches, when the ports are closed, is not so much more complete in vessels with one deck than with those with two decks as to counterbalance the advantages in point of exterior ventilation which ships of the line enjoy. To show to what an extent this crowding of the sleeping berths takes place, it is sufficient to state that the usual space between the suspending points (clues) of the hammocks is from fourteen to eighteen inches; so that when they are extended by the beds, the bodies of their occupants are in contact. "When at sea, with a watch on deck, the accumulation and pressure are reduced by a half, but when in secure harbour, 500 men perhaps sleep on one deck, their bodies touching each other over the whole space laterally, and with very little spare room lengthways." Under ordinary circumstances, this crowded state of the decks does not appear to exercise any very deleterious influence on health, but its influence on those already attacked by diseases of an infectious character or accompanied by great debility and depression, must not be lost sight of in estimating the effect of a seafaring life on health.

Cleanliness both of the vessels and of the person, we need not say, is well attended to in the navy. There is little doubt, indeed, that in one respect it is carried too far. We allude to the practice of frequently washing the several decks, a practice censured by Dr. Wilson, and condemned by experience. There can be no doubt that this practice keeps the decks of a vessel in a constant state of dampness, which must prove highly prejudicial to health. Cleaning the decks, with the exception perhaps of the upper deck and upper gun-deck in ships of the line, by *dry stoning* is certainly very much to be preferred to repeated washings. The moist state of atmosphere kept up by this practice must be borne in mind in summing up the injurious influences brought to bear on the sailor. Two or three striking examples of the effect of moisture in inducing the scurvy are related in Parry's Voyages, and we are ourselves indebted to an old naval officer for an apt illustration of its effects drawn from a comparison of the health of two ships' crews, the one English and the other French, occupying for some time the same station. In the English vessel the decks were constantly washed, in the French vessel they were dry-rubbed. The scurvy was rife in the one, while not a single case of it existed in the other. In all other respects the English vessel was at least on a par with the French one.

Ventilation, which has already been incidentally mentioned, has not yet been carried to a high degree of perfection on board ship, nor are its principles as yet thoroughly understood as applied to buildings on land. Wind-sails, which have hitherto been the chief means of ventilation employed, are objectionable, inasmuch as they are inoperative in calms and in wet weather, and even with strong breezes and in dry weather they ventilate but a small portion of the decks. The apparatus lately invented by Captain Warrington is free from these objections, but is yet far from perfect. There is no doubt that it will admit of considerable improvement.

The last iufluence noticed in the report as brought to bear on the health of seamen is amusement and intellectual culture. We need not remind the professional reader of the vast importance of this influence to the bodily health, and we congratulate ourselves that it has been at length so successfully brought to bear on the minds of our seamen. Not the least interesting fact mentioned in the introduction to these reports is the following: That "by an admiralty order, dated August 1838, libraries are directed to be established in each of Her Majesty's ships, for the use of the crew, furnished at the public expense, and placed in charge of the schoolmaster. The books, amounting to 270 volumes for larger, and 100 for smaller ships, exclusive of bibles, are judiciously chosen, with the view of combining amusement and instruction, and making the first subsidiary to the last. Besides the accomplished men now appointed to instruct the junior officers, it is further directed by an order from the admiralty, May 1837, that a fit person shall be appointed to give elementary education, comprehending reading, writing, and arithmetic, to the sailor boys and other seamen and marines who may require it." Though last in order, this is by no means least in importance, amongst those regulations of the admiralty which have tended so strikingly to improve the health and increase the efficiency of our marine.

We cannot quit this introductory chapter of the reports, without adverting to the vast improvement which has taken place of late years in the comfort and health of our seamen, and in the consequent efficiency of our naval service. "Formerly," says the report, "a ship of war was on many accounts an object of aversion," and well it might be. "A diet consisting of very salt beef, biscuits long baked, and puddings made of salt suet and flour..... Water so putrid and offensive, often so thick and green from vegetable admixture and decomposition, and emitting so strongly the fetor of rotten eggs, as to disgust at once the sense of smell and of taste;" the best and almost only luxury of the seamen an exorbitant allowance of spirituous liquors, at sea as on land the fruitful source of disease, and misery, and insubordination, and crime in all its shapes; the ship itself damp, filthy, ill-ventilated; the air of the wells so contaminated that fatal asphyxia was by no means a rare consequence of inhaling it; personal cleanliness neglected, the clothing insufficient; but little care taken to amuse the mind and none to instruct it: add to all these discomforts, a discipline not merely strict but severe, and punishment too often inflicted at the instigation of momentary passion—and we have a faithful picture of the naval service in former years. But these hardships and deprivations, painful as they are to contemplate, formed nevertheless but a small part of the miseries of our seamen. Disease was wanted to give the finishing touch to the picture, and convert the scene of hardship and deprivation into one of thrilling horror. "Scurvy, putrid ulcer, malignant dysentery, and fever, allied to that of gaols, suddenly swept off the greater portions of many ships' crews, and well-nigh depopulated fleets." The first of these diseases alone has sufficed to place a well-manned vessel at the mercy of the winds and waves. Witness Lord Anson's ship, the Centurion, in 1742, "the crew so weakened by scurvy, that only eight men were capable of doing duty, and these so reduced in health, that had the ship been compelled to keep the sea a very few days longer, it would not have been possible to have brought her to an anchor at Juan Fernandez, and she must have gone adrift in the Pacific Ocean, as once happened to a Spanish ship in the same ocean under the same circumstances."* So dreadful was the mortality at sea from this one cause alone, that the description given by a Portuguese historian of the early expeditions of that nation in pushing their discoveries on the African coast in search of India, seems but a warrantable hyperbole. "If the dead," says he, "who had been thrown overboard between the coast of Guinea and the Cape of Good Hope, and between that Cape and Mozambique could have tombstones placed for them, each on the spot where he sank, the whole way would appear one continued cemetery." In large fleets the mortality was not less frightful than in single ships. Sir Richard Hawkins, the great navigator who lived in the time of Elizabeth and her successor, relates that in the course of twenty years, he "had known of 10,000 seamen" (a number short by only about 4000 of all who served in the fleet which conquered the Spanish Armada) "having perished by scurvy alone." This fearful mortality is not a story merely of the olden time, for even so late as the year 1780, Sir Gilbert Blane found that in a fleet manned with between 7000 and 8000 seamen, the mortality had been one in seven the preceding year.

Turn we from this harrowing picture of hardship, disease, and death, to that which is presented by our ships of war in more recent times. A diet at once abundant, and varied, and wholesome, a plentiful supply of pure water, cleanliness carried almost to a fault, constant attention to the ventilation and drying of the vessel, an ample supply of warm clothing; add to these bodily comforts so beneficial to health the excellent diet and superior medical treatment provided for the sick, and the arrangements lately adopted for amusing and instructing the mind, as well as the improved discipline and admirable limitations set to the capricious exercise and abuse of authority; and we may safely affirm that a ship of war, instead of being as formerly "an object of aversion," cannot fail to present many attractions to the seaman; and sure we are that it is by arrangements such as these, and not by a vain attempt to

* Sir Gilbert Blane, Health of Seamen, prefatory discourse.

compete with the merchant service in the wages offered to the sailor, that our navy will be manned with hardy frames and willing spirits, ready now as heretofore to brave every danger in defence of the country which shows itself thus careful of their health, comfort, and improvement.

The success which has attended these well-directed attempts to preserve and improve the health of our seamen is too well known to require any lengthened remarks. The late expeditions to the Polar seas supply the best commentary on their efficacy. The share which each of the several arrangements which we have specified has had in producing the general result may admit of some controversy; and it might be interesting to enquire how much of the good effected is due to the improved medical treatment of the sick. That this has played no unimportant part in achieving the high state of health now enjoyed in the navy, no one, but especially no medical man, will be disposed to doubt; still it is to hygienic arrangements, properly so called—to improvements in diet, ventilation, clothing, &c.—that the result is mainly attributable. In a national point of view, and as a question of finance, the health of the navy is one of the most important which can occupy our attention; and the success which has attended the methods employed to preserve it, may well raise expectations of still more extensive benefits to be conferred on other sections of the community, whose interests in this respect have been hitherto so grossly neglected.

If it be true, as it undoubtedly is, that these improvements in health have alone sufficed to double the effective force of our navy, to make one ship for all purposes of navigation and warfare equivalent to two of equal force, to enable a vessel to keep the sea for twice or thrice the time which was possible some fifty years ago; if it be true that at the old rate of mortality, all Europe could not have furnished the seamen necessary for our defence and safety during the great revolutionary war,*—then it is a mere waste of words to prove that public health is a national blessing, and the neglect of it a calamity and a curse. Motives of economy alone must influence government to spare no exertion by which the health of our navy may be promoted and secured; for it is clear that if one man can be made to do the work of two, the expense of one man must be saved; and, if this unit be multiplied by the number of men actually employed in our navy, we have a product which can scarcely fail to strike the mind of the economist. But this by no means represents the whole saving to the nation which accrues from such improvements in the health of our seamen; we must add to this all the expense formerly incurred in raising men to supply the place of those whom disease had cut off or rendered useless. But we have not yet exhausted all the arguments which might be urged in favour of a scrupulous and watchful care of the health of our navy. It is not merely that our force has been doubled and our expenses diminished by more than a half, but each individual employed must be made more effective by having his health carefully preserved to him, and his life prolonged to gain increased experience and aptitude for his employment.

We trust that the encouraging example afforded by the navy will not be lost upon those who have it in their power to extend these provisions for the preservation of health to the other branch of the public service,

* Sir Gilbert Blane, Health of the Navy.

and to the community at large. The necessity may not appear so pressing, nor the advantage so obvious, but the one is as real as the other is substantial. Sickness and death in our fleets formerly cost an extravagant outlay of public money; do the pestilential diseases and frightful mortality of some of our large cities cost the public nothing? Do fever hospitals and workhouse infirmaries, and pauper burials, and widows, and orphans, and cripples—the victims of diseases, which might have been prevented but cannot be cured—cost nothing? If but a small fraction of the money expended on objects such as these had been employed in constructing sewers and widening streets, and providing places of recreation in the centre of our crowded cities; in letting the light of heaven into their dark and gloomy recesses, and the sweet breath of heaven into their foul and reeking habitations, we should not have now to lament over such scenes as those which the great manufacturing capital of the north has lately disclosed. The preservation of the public health is economy in every sense. It not only saves money, but it makes it. It substitutes men for children, productive for unproductive labourers. It supplies a country with its best riches and its cheapest defence—the arm strong to labour and to fight.

But these reflections are leading us too far from our subject. The report comprises three commands—the South American, the West Indian and North American, and the Mediterranean and Peninsular, and extends over seven years, namely, from 1830 to 1836, inclusive. The diseases, invaliding, and mortality for each year are represented in four tables, of which the first shows "the total number of cases; the number of all diseases and injuries in classes; the number of cases sent to hospital, invalided, and dead; with the ratio of each per thousand of mean strength." The second table exhibits "the total number of cases; the number of all diseases and injuries in classes; the number invalided and dead; with the ratio of each per thousand attacked." The third table represents "the number of cases of principal diseases and injuries; the number sent to hospital, invalided, and dead; with the ratio of each per thousand of mean strength." The fourth table shows "the number of frequent, but not often, fatal diseases; the number sent to hospital, invalided, and dead, with the ratio of each per thousand of mean strength." The diseases included in this table are superficial inflammation of the extremities, rheumatism, catarrh, and diarrhœa. The first two tables are thus subdivided—febrile diseases, nervous diseases, cachectic diseases, and local diseases. As we have already stated, Cullen's nosology is that adopted in the navy. We are sorry for it, as we believe it to be based too much upon mere hypothesis, and to be very badly adapted to statistical reports. For this purpose we greatly prefer the arrangement adopted in the Army Reports by Major Tulloch; and it would certainly have been much more convenient to have been able to compare the Navy Reports with the able statistical documents which preceded them. On glancing over these first two tables, we observe such anomalies as the following: pulmonary consumption classed with "hemorrhages with fever," and forcibly separated from inflammatory affections of the lungs; diarrhœa placed among spasmodic diseases, as a sub-class of nervous diseases, whilst dysentery occupies a place among febrile diseases, because, forsooth, it is a flux, accompanied by fever; then again jaundice, which most medical men regard as an affection of the liver, or its excreting ducts, is marshalled with syphilis and scrofula, as a cutaneous disease, under the general head of cachectic diseases. Emaciation of the body is made to "include intestinal worms, and certain *obscure consumptive diseases* of the alimentary canal," whilst "swellings and dropsies" form a sub-class of cachectic diseases, though "tumours, including aneurism, varicose veins, and bubo," are "local diseases." We are far from wishing to lay the obvious defects of this sort of classification to the charge of the compiler of these tables. He has been obliged, no doubt, to frame his tables from the reports submitted to him, and these unfortunately follow the nosology of Cullen; which, bad as it is, was, we believe, the best at the period when the plan of these medical returns was introduced. The third table happily dissolves, to a certain extent, this unnatural alliance between diseases which have so little in common, except the faulty hypothesis which first brought them together, and we are presented with a catalogue of the principal diseases, which we copy for the benefit of those who may wish to know what those principal diseases are. The list is as follows: Fevers, inflammation of the lungs, inflammation of the liver, organic diseases of the brain, consumptive diseases of the lungs, expectoration of blood, dysentery, inflammation of the stomach and bowels, delirium tremens, syphilis, gonorrhœa, ulcers, wounds, and accidents.

The tables for each year are followed by a few pages of remarks, either suggested by the tables themselves, or by the several journals and reports from which they have been compiled, and the results obtained from the seven successive years are thrown into four general tables, arranged like those of the individual years, and followed like them by general remarks. Towards the end of the volume an attempt is made to ascertain the "influence of forms of ships on health," by constructing tables similar to the first of those already described for vessels of the first class (seventy-two guns and upwards), for the second class (frigates), the third class (corvettes), and the fourth class (steamers). The report is concluded by tables presenting the individual facts, from which the aforesaid tables have been constructed, and specifying the name and force of the several ships employed.

Having thus described the contents of the reports, the next question is, how shall we make use of the extensive materials contained in it, so as to place the greatest possible amount of information in the hands of our readers? After much reflection, we have come to the resolution to disappoint them for the present of some of the instruction which they perhaps expected to obtain, and to postpone an analysis of much of the most interesting matter comprised in this volume until the remaining reports, which, we are glad to find, are in a forward state of preparation, make their appearance. When all the reports are before us, we propose making a careful analysis of all the information they contain with regard to some of the principal diseases, comparing one command with another, and embodying in one account all the knowledge to be gleaned, not from the tables merely, but from the text of the several reports. If we have leisure for so grave an undertaking, we shall then compare the health and diseases of the navy with those of the army; but, that we may do this, it will be necessary to remodel the navy reports, so as to be enabled to compare them with the more simply and scientifically arranged reports of Major Tulloch. After this explanation of our intention, it is obvious that our present notice must be considered merely in the light of an introduction to the more laborious articles which we have in store, and we shall accordingly content ourselves with giving such an account of the several commands as may throw light on the discussions into which we shall have to enter.

*The South American Command.* "The South American Naval Command embraces a great extent of coast and cruising ground. It extends on the east side of the peninsular continent, from Para to Cape Horn, and on the west from Cape Horn to Panama, and thence to California. It reaches from the 30th degree of north to the 58th degree of south latitude, across the Pacific, from the equator to 58 degrees south, across the South Atlantic; and from Cape St. Royal in the 35th, to California in the 120th degree of west longitude. It is therefore exposed to almost all degrees of atmospheric temperature, from the highest to the lowest, with the ordinary meteoric agencies thence arising. Many of the ports and places of anchorage, and adjacent territories, differ from each other in almost every manner and degree. In relative position and nature and extent of exposure, in respect of contiguous land, its general form, and distance, and influence on wind and rain, and in the soil and products of the soil, there is great, sometimes extreme difference in the various places resorted to by British ships. The places principally frequented are Rio de Janeiro, Buenos Ayres, Bahia, Panambuco and Para, Valparaiso, Callao, Coquimbo, Panama, and San Blas. In some of them art has been busy and effective; large towns have been built, and the ground has been cleared and cultivated in the neighbourhood of the towns, and on the margins of the bays and harbours; in others, beyond rearing houses and stores, things remain very nearly in their natural condition. Hence extensive marshes are in close contact with many of them; while in others, but more from natural formation than the labours of man, dry land, and little productive of vegetable matter, abounds. The places named are, with three exceptions, situate within the tropics, some near their external limits, some close to the equator, and others at different intermediate points; yet with all such difference in position, soil, products of soil, and climatorial heat, the inhabitants of the shores of this vast continent, whether permanent or occasional, enjoy a high and a singularly uniform degree of health.......It is peculiar to this command, that during peace the ships are employed altogether on an alien coast. Along its whole extent, and nowhere within the limits of the command, excepting the recent small settlement at the Falkland Island, is there any British possession. Hence there are no hospitals for the reception of British sailors; the want of which, though it may not materially and directly affect life, increases the necessity for invaliding. Many men labouring under mere chronic forms of disease might be cured in hospital, and saved to the squadron, who cannot be restored to health on board ships."

The aggregate numerical force of the seven years was 17,254, giving an average of 2,465 per annum. Taking one year with another, the number of vessels employed was twenty-five, of which one was a ship of the line, five or six frigates large and small, and the remainder sloops and brigs, including packets. It has already been stated, that the inhabitants of the shores of the continent included in the South American command enjoy a high state of health. The same remark applies to the naval forces employed, the mortality being 7·7 per 1000, a mortality much inferior to that of persons of corresponding ages in the United Kingdom.

*West Indian and North American Command.* This command "extends from the equator to the 60th degree of north latitude, and embraces every British possession, continental and insular, from Guaiana to Cape Charles on the coast of Labrador. It consequently contains almost every degree and variety of climate, dependent on temperature, atmosphere, and soil, and their reciprocal relations, many of them being of course common, some of them in a great measure peculiar." The great extent of this command, and the variety of climate which it embraces, render it very difficult to draw conclusions of any value concerning the influence of climate on health. To arrive at any satisfactory result it would be necessary to separate the West Indies from North America, and to group the Bahama islands and the Bermudas with the former. As this cannot be done, we must content ourselves with such mixed results as can be obtained from the tables. The mean annual force amounted to 3,326, and the number of vessels of all descriptions, many of which were of small size, to forty-seven. The annual rate of mortality in this command, on an average of seven years, from disease alone was 18·1 per 1000 of mean strength, from fever alone 11·1.

*The Mediterranean and Peninsular Command.* "This command embraces the seas and shores of the Mediterranean and Gibraltar. It extends over less space than some other commands, comprising fewer degrees of latitude, and is less exposed to extreme difference in degrees of temperature; it does not include more than 12 degrees, those, namely, from the 32d to the 44th north. As respects geographical position, therefore, the term 'temperate' has been especially applied to it, and it has obtained high reputation for its influence on health. Yet the difference between the south and north shores, as regards temperature, is often great, especially in winter; the vicissitudes on the north coast are sudden and violent, and the effects of the climate either in the prevention or remedying of some diseases, particularly those affecting the lungs, are perhaps not so great as they have often been represented. The sirocco, south-east wind, blowing from the African continent, is singularly depressing, suddenly producing such languor, oppression, and feelings of feebleness, as neither its temperature, nor other appreciable quality can account for. It seldom continues to blow during many consecutive days; what its ultimate influence on health might be, if long-continued, cannot be ascertained, though there can be little doubt that it would be highly prejudicial; but during the period it lasts, notwithstanding the distressing power it exercises over sensation, it does not appear to injure the organic powers of life. Its influence is most felt near the coast of Africa, but it is often powerful at Malta and Sicily, and sometimes reaches the north shores of the Mediterranean. Malta, on account of its central situation, its arsenals, and the excellence of its harbours, is the principal naval station. During nine months of the year, the temperature is moderate, the weather being generally clear and fine, which, with the excellence of fresh meat and vegetables procured there, produces highly beneficial effects on the health, comfort, and efficiency of the naval force employed in the Mediterranean. The other three months are hot, sometimes in a high degree; but either because it is not sufficiently long continued, or because there is not much of morbific material for it to act on, the heat has not, except on some very rare occasions, proved injurious to health to any extent. Every anchorage in the circuit of the Mediterranean is visited, and more or less frequented, by ships of war. The ports most resorted to are Malta, Smyrna, various Greek and Levantine islands, and Gibraltar, the extent to which they are occupied differing much at different periods.......The numerous ports and places embraced by this command differ greatly as to extent, exposure, and physical structure, and considerably in regard to external heat; they therefore act very differently on the health of ships' companies resorting to them; but on the whole, and apart from some rare but severe epidemics which have affected some of them, their influence, combined with that of the sea-climate, is very favorable.......The naval force employed on the coasts of Spain and Portugal is included in the Report for the Mediterranean."

The mean annual strength of the squadron in this command was 7,958, number of vessels forty-five to fifty-six, including many ships of the line and large frigates. The mean annual mortality in this station was, from disease alone, 9·3 per 1000 of mean strength. Comparing the mortality of the three commands included in the report, we find the result as follows:

| | Disease and accident. | Disease alone. |
|---|---|---|
| South American ....... ........ | 8·9 | 7·7 |
| West Indian and North American .. | 19·6 | 18·1 |
| Mediterranean and Peninsular .... | 11·1 | 9·3 |

The South American command, therefore, is the most healthy, next to that the Mediterranean and Peninsular; and, by far the most unhealthy, is the West Indian and North American.

We do not intend at present, as we have already stated, to enter into the subject-matter of these reports at greater length. We must remind our readers a second time that the present sketch is merely introductory to a more minute and careful abstract, which we hope to render both interesting and instructive. We shall notice the forthcoming reports in the same way, and reserve ourselves till we have them all at the same time under our eye. In the meantime we dismiss the present volume with much commendation to the compiler for the industry and intelligence he has displayed, but with regret that the adoption of a defective nosological system has impaired the value of his labours.

---

